# Chromatin-derived histone proteins from *Schmidtea mediterranea* function as innate antimicrobial effectors

**DOI:** 10.3389/fcimb.2026.1816580

**Published:** 2026-04-20

**Authors:** Devika Nampoothiri, Riddhi Bhardwaj, Nimish Deshpande, Prasad Abnave

**Affiliations:** 1 BRIC-National Centre for Cell Science, Savitribai Phule Pune University, Pune, Maharashtra, India; 2 Regional Centre for Biotechnology, NCR Biotech Science Cluster, Faridabad, India

**Keywords:** antibacterial, histone, infection, planarian, *Schmidtea mediterranea*, *Staphylococcus aureus*

## Abstract

Planarians display remarkable resistance to bacterial infection, including infection by *Staphylococcus aureus*, yet the molecular mechanisms underlying this antibacterial capacity remain poorly defined. Histones are highly conserved chromatin-associated proteins traditionally known for their structural role in nucleosome organization; however, studies in diverse organisms have revealed that histones can function as antimicrobial effectors when released extracellularly. Whether histone proteins contribute to antibacterial defense in planarians has not been investigated. Here, we identified core histone proteins (H1/H5, H2A, H2B, H3, and H4) from the transcriptome of *Schmidtea mediterranea* through comparative sequence analysis using reference histones from *Homo sapiens*, *Drosophila melanogaster*, and *Caenorhabditis elegans*. Domain architecture and phylogenetic analyses confirmed their evolutionary conservation. Core histones were isolated from planarian nuclei by acid extraction and validated by SDS-PAGE and immunoblotting. Functional assays revealed that histone-enriched extracts isolated from planarian nuclei exhibit selective and dose-dependent antibacterial activity against Gram-positive bacteria, with the strongest inhibition observed for *S. aureus*, whereas minimal effects were detected against Gram-negative species such as *Escherichia coli*. Histone treatment induced membrane permeability and bacterial death, as demonstrated by SYTO9/propidium iodide staining and confocal microscopy. Importantly, protease treatment and heat denaturation abolished antibacterial activity, confirming that the effect depends on intact protein structure. Collectively, our findings demonstrate that histone-enriched chromatin extracts from *S. mediterranea* possess intrinsic antibacterial activity with preferential efficacy against Gram-positive bacteria, supporting a model in which chromatin-derived histone proteins represent conserved components of innate immune defense in planarians.

## Introduction

The freshwater planarian *S. mediterranea* is widely used as a model for regeneration and stem cell biology. In addition to its regenerative capacity, this organism survives in microbe-rich aquatic environments and displays robust innate immune responses (Kangale et al., 2021; Maciel and Oviedo, 2018; Peiris et al., 2014). Planarians have been reported to exhibit notable resistance to bacterial pathogens, including *Staphylococcus aureus*, yet the molecular mechanisms underlying this antibacterial capacity remain incompletely defined (Abnave et al., 2014; Torre et al., 2017). Understanding the effector mechanisms that contribute to pathogen control in planarians may provide insight into evolutionarily conserved strategies of host defense.

Innate immunity employs diverse mechanisms to eliminate invading microorganisms. Beyond classical antimicrobial peptides, emerging evidence indicates that certain intracellular proteins can acquire antimicrobial functions when released into extracellular environments. Histones are highly conserved, positively charged proteins that form the structural core of nucleosomes and regulate genome organization. Increasing evidence from vertebrate systems demonstrates that extracellular histones exert direct bactericidal activity, including during the formation of Neutrophil extracellular traps, where chromatin-associated histones contribute to pathogen killing (Fu et al., 2020; Hoeksema et al., 2016; Jodoin and Hincke, 2018; Li et al., 2022; Muñoz-Camargo and Cruz, 2024; Zeng et al., 2024). These observations suggest that chromatin-derived proteins may function as conserved antimicrobial effectors across metazoans.

Despite the evolutionary conservation of histone proteins, their potential contribution to antibacterial defense in planarians has not been explored. Given the reported resistance of planarians to *S. aureus* and the cationic properties of histones that facilitate interaction with negatively charged bacterial membranes, we hypothesized that planarian histones may possess intrinsic antibacterial activity. To test this hypothesis, we identified core histone homologs in *S. mediterranea* through comparative sequence analysis and phylogenetic reconstruction using reference histones from *Homo sapiens*, *Drosophila melanogaster*, and *Caenorhabditis elegans*. We subsequently isolated histone proteins from planarian nuclei and assessed their antibacterial activity against representative Gram-positive and Gram-negative bacterial species.

## Materials and methods

### Planarian culture

The clonal asexual strain of *S. mediterranea* was maintained in 1× Montjuïc water (1.6 mM NaCl, 1.0 mM CaCl_2_, 1.0 mM MgSO_4_, 0.1 mM MgCl_2_, 0.1 mM KCl, 1.2 mM NaHCO_3_ prepared in sterile distilled water) at 20°C in the dark. Animals were fed goat liver once per week and rinsed daily. Prior to experiments, planarians were starved for at least 7 days.

### *In silico* identification of planarian histone proteins

Reference histone protein sequences from *Homo sapiens*, *Drosophila melanogaster*, and *Caenorhabditis elegans* were retrieved from UniProt. The *S. mediterranea* transcriptome dataset was downloaded from PlanMine in FASTA format (Rozanski et al., 2019). tBLASTn searches were performed locally using reference histone proteins against the planarian transcriptome to identify putative homologs. Candidate nucleotide sequences were translated using the ExPASy Translate tool. Reciprocal BLAST analysis was performed to confirm identity. Conserved domains were verified using InterPro.

### Phylogenetic analysis

Histone sequences from planarian and reference organisms (*Homo sapiens*, *Drosophila melanogaster*, and *Caenorhabditis elegans*) were aligned using ClustalW implemented in MEGA11. Phylogenetic trees were constructed using the Maximum Likelihood method. Bootstrap analysis was performed with 100 replicates to assess node support.

### Acid extraction of histone proteins

Histones were extracted as described previously with minor modifications (Shechter et al., 2007). Briefly, 5–10 starved planarians were minced on ice in cold PBS containing 0.5% Triton X-100, 1 mM PMSF, 1× protease inhibitor cocktail, and 0.02% sodium azide. The detergent containing lysis buffer selectively disrupts cellular membranes while leaving nuclei intact, allowing enrichment of the nuclear fraction by centrifugation prior to acid extraction of chromatin-associated proteins. Homogenates were centrifuged at 10,000 rpm for 10 min at 4°C. The supernatant was retained as the cytoplasmic fraction. The nuclear pellet was resuspended in 400 µL of 0.4 N H_2_SO_4_ and incubated for 1 h at 4°C with gentle rotation. Following centrifugation (16,000 × g, 10 min, 4°C), the acid-soluble supernatant containing histones was collected. Histones were precipitated by adding trichloroacetic acid (132 µL per 400 µL extract) and incubating at −20°C for 1 h. Samples were centrifuged (16,000 × g, 10 min, 4°C), and pellets were washed twice with chilled acetone. Pellets were air-dried briefly at room temperature and resuspended in 50 µL nuclease-free water. Extracts were stored at −20°C.

### Protein quantification, SDS–PAGE, and coomassie staining

Protein concentration was determined using the Bradford assay according to the manufacturer’s instructions. Absorbance was measured at 600 nm, and concentrations were calculated using a BSA standard curve. Histone extracts were resolved on 15% SDS–PAGE gels. Samples were mixed with 6× loading dye, denatured at 95°C for 10 min, and electrophoresed at 70–75 V. Gels were stained with Coomassie Brilliant Blue for 1 h and destained overnight. Protein bands were visualized using a gel imaging system.

### Western blotting

Proteins were transferred from SDS–PAGE gels to PVDF membranes at 110 V for 1.5 h at 4°C. Membranes were blocked in 5% skimmed milk in TBST for 2 h at room temperature and incubated overnight at 4°C with primary antibodies: anti-histone H3 (CST # 4499, 1:2000), anti-histone H4 (CST # 13919, 1:5000), and anti-histone H2A (Abcam # 18255, 1:5000). After washing, membranes were incubated with HRP-conjugated anti-rabbit IgG secondary antibody (CST # 7074, 1:10000) for 2 h at room temperature. Signals were detected using enhanced chemiluminescence substrate and imaged using a chemiluminescence detection system.

### Bacterial strains and culture conditions

The following bacterial strains were used. Gram-positive: *Staphylococcus aureus* (ATCC 29213), *Streptococcus pneumoniae* (ATCC 6303), *Enterococcus faecalis* (ATCC 12399). Gram-negative: *Escherichia coli* (ATCC 25922), *Pseudomonas aeruginosa* (ATCC 10145), *Klebsiella pneumoniae* (NCMR MCC 2451). Bacteria were cultured in Nutrient Broth at 37°C to mid-logarithmic phase. Cell density was adjusted to 1 × 10^8^ CFU/mL in phosphate-buffered saline (PBS, pH 7.4) prior to antibacterial assays.

### Antibacterial activity assay

For screening assays, 50 µL of histone extract (1 µg total protein) was added to 96-well plates, followed by 10 µL bacterial suspension (1 × 10^8^ CFU/mL). Samples were incubated for 2 h at room temperature to allow interaction. Subsequently, 140 µL Nutrient Broth was added, and plates were incubated at 37°C. Bacterial growth was monitored by measuring OD_600_ hourly for 6 h. Gentamycin (0.1 µg) served as a positive control, BSA (1 µg) as a protein control, and untreated bacteria as a growth control. Experiments were performed in at least three independent biological replicates. To assess dose-dependent antibacterial activity, histone extracts were tested against *S. aureus* at concentrations of 0.2, 0.4, 0.6, 0.8, and 1 µg per well. Equivalent concentrations of BSA were used as controls. Bacterial growth was monitored as described above.

### Bacterial viability assay

Bacterial viability was assessed using the LIVE/DEAD^TM^ BacLight^TM^ Bacterial Viability Kit (Invitrogen # L7012) according to the manufacturer’s instructions. After 4 h of histone treatment, bacteria were pelleted (13,000 rpm, 2 min), washed with PBS, and stained with SYTO9 and propidium iodide for 30 min in the dark at room temperature. Samples were mounted on glass slides and imaged using a Zeiss LSM 880 confocal microscope. Heat-killed bacteria served as a positive control for membrane-compromised cells.

### Protein dependency assays

To determine whether the antibacterial activity of planarian histone extracts was protein-dependent, enzymatic digestion and heat-denaturation assays were performed prior to antibacterial testing. Histone extracts (1 µg) were subjected to - a) Proteinase K treatment by incubation with 100 µg/mL Proteinase K at 37°C for 1 h, followed by heat inactivation at 95°C for 10 min. b) Heat denaturation by incubation at 95°C for 15 min. Treated and untreated samples were tested against *S. aureus* using the growth inhibition assay described above.

### Statistical analysis

All experiments were performed using at least three independent biological replicates unless otherwise stated. Data are presented as mean ± standard deviation (SD). Statistical analyses were conducted using GraphPad Prism software. Unpaired two-tailed Student’s t-test was used for comparisons between two groups. Differences between groups were evaluated using one-way ANOVA followed by Tukey’s multiple comparison test. A p-value < 0.05 was considered statistically significant.

## Results

### Identification and evolutionary conservation of planarian histones

**Figure 1:**
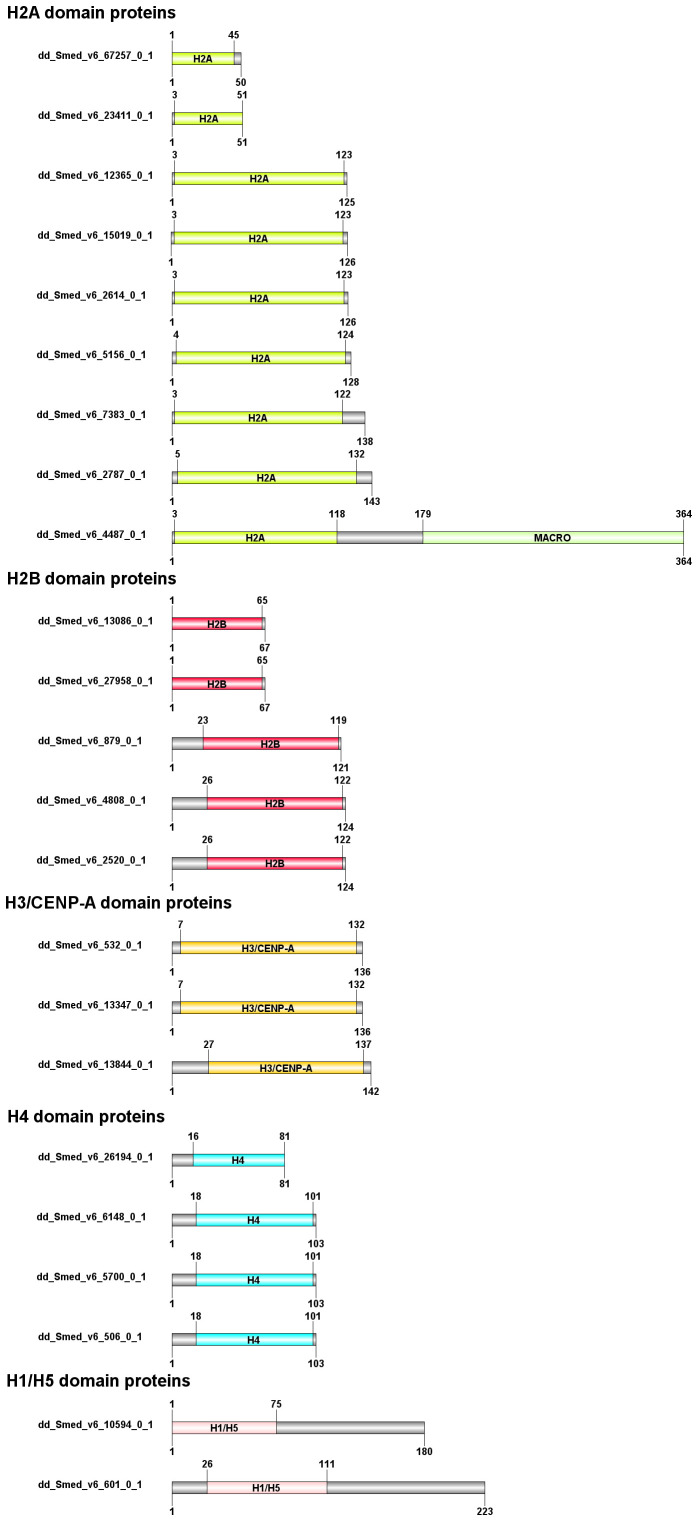
Identification of planarian histone proteins. Predicted histone proteins in *S. mediterranea* with H2A, H2B, H3/CENP-A, H4, and H1/H5 domains.

To identify histone homologs in *S. mediterranea*, the transcriptome was searched using reference histone sequences from human, *Drosophila melanogaster*, and *Caenorhabditis elegans*. Multiple transcripts corresponding to the five major histone classes (H1/H5, H2A, H2B, H3 including CENP-A, and H4) were identified (**Figure 1**). Reciprocal BLAST confirmed their identity, and InterPro analysis verified the presence of conserved histone fold domains in each case.

The predicted H2A proteins were approximately 50–145 amino acids in length and contained the canonical H2A domain. One H2A variant possessed an extended C-terminal MACRO domain, consistent with a macroH2A-type protein. H2B proteins ranged from ~70–125 amino acids and carried the conserved H2B fold domain. H3 proteins (~135–140 amino acids) displayed the expected H3/CENP-A architecture, while H4 proteins (~80–105 amino acids) retained the highly conserved H4 core domain. Linker histones (H1/H5) were longer (~180–220 amino acids) and showed the characteristic tripartite organization with a central globular domain. Overall, these analyses indicate that *S. mediterranea* possesses a complete set of structurally conserved repertoire of histone proteins.

**Figure 2:**
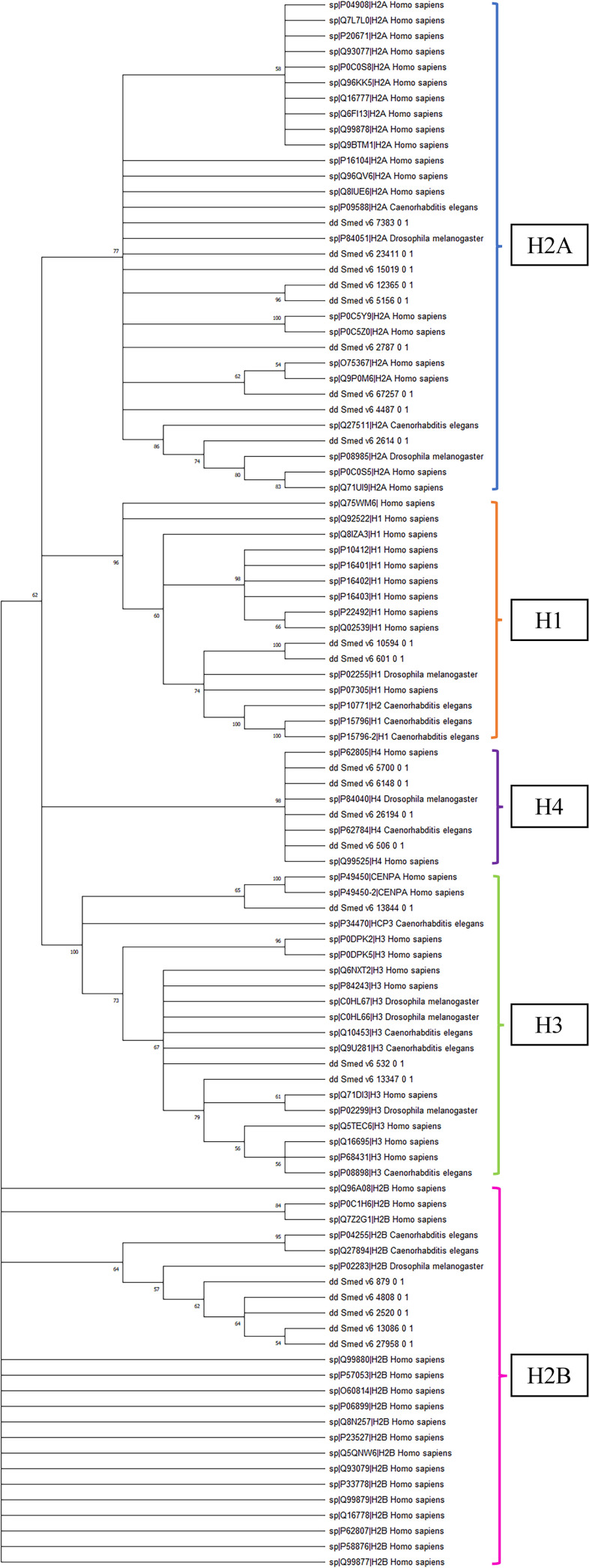
Phylogenetic analysis of planarian histone proteins. Phylogenetic tree of 101 histone proteins (16 H1, 32 H2A, 25 H2B, 20 H3, and 8 H4) from four species (*S. mediterranea*, *H. sapiens*, *D. melanogaster*, and *C. elegans*) constructed with 100 bootstraps.

To assess evolutionary relationships, a phylogenetic tree was constructed using the Maximum Likelihood method, incorporating histone sequences from *S. mediterranea*, *Homo sapiens*, *C. elegans*, and *D. melanogaster*. The resulting unrooted tree revealed clear clustering according to histone class rather than species (**Figure 2**). Planarian sequences consistently grouped with their respective histone-type orthologs. H2A, H2B, H3, H4, and H1 proteins each formed well-supported clades containing both vertebrate and invertebrate representatives. This clustering pattern reflects the strong evolutionary conservation of histones across metazoans.

### Extraction and validation of planarian histone proteins

**Figure 3:**
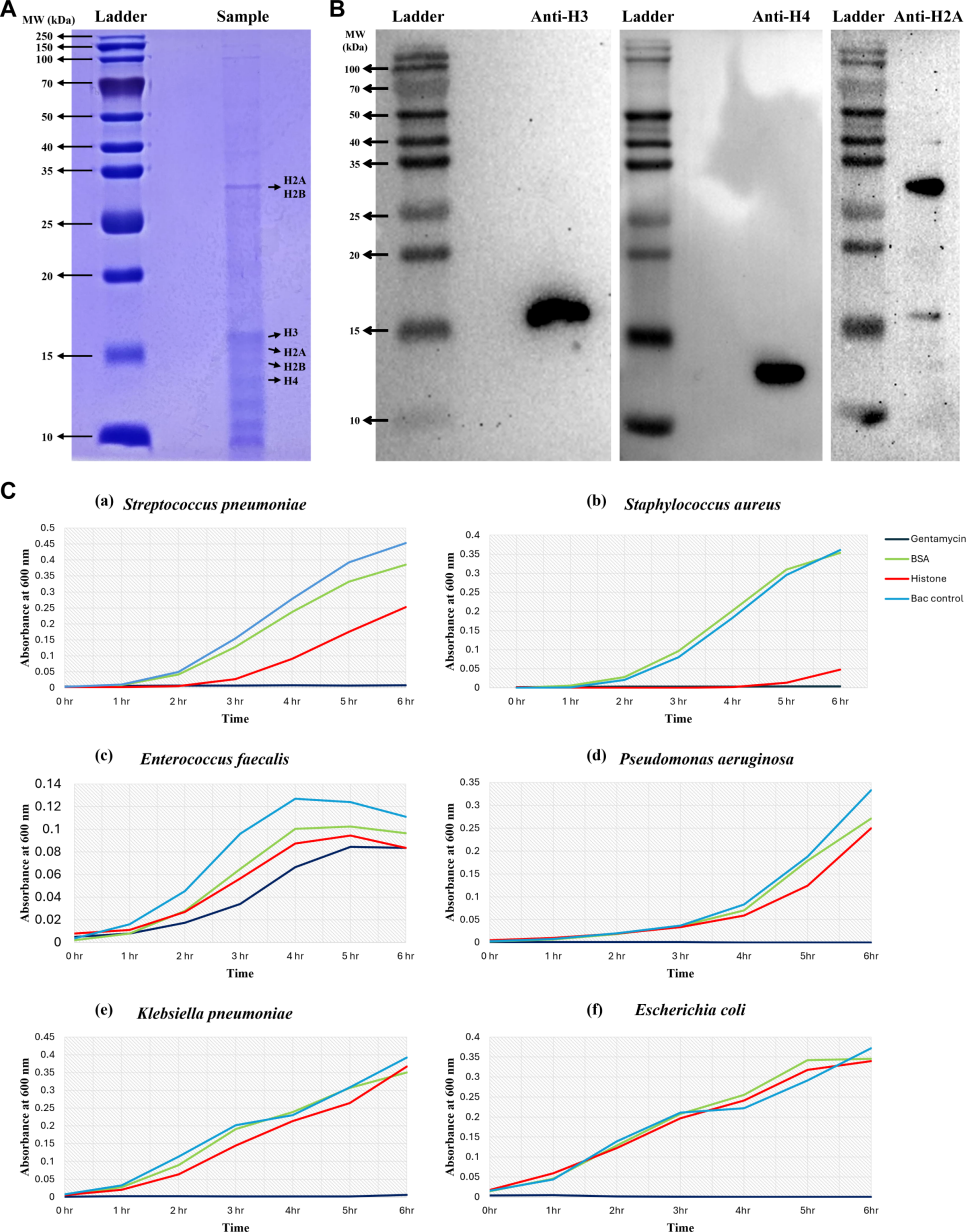
Planarian histone extraction and antibacterial activity. **(A)** Coomassie-stained SDS–PAGE showing bands of planarian histone proteins extracted by acid extraction method. Major bands corresponding to core histone proteins (H2A, H2B, H3, and H4) are indicated based on expected molecular weights. **(B)** Western blot analysis of the extracted fraction using anti-histone H3, H4, and H2A antibodies. **(C)** Antibacterial activity of planarian histone extracts (1 µg) against Gram-positive (*S. aureus*, *S. pneumoniae*, *E. faecalis*) and Gram-negative (*E. coli*, *P. aeruginosa*, *K. pneumoniae*) bacteria. Gentamycin (0.1 µg) served as a positive control, BSA (1 µg) as a protein control, and untreated bacteria as growth control.

Histones were isolated from *S. mediterranea* by acid extraction of purified nuclei followed by TCA precipitation. Protein concentration was determined by Bradford assay. SDS–PAGE analysis showed prominent low-molecular-weight bands between ~10 and 17 kDa (**Figure 3A**), consistent with the expected sizes of core histones H4 (~12 kDa), H2B (~14 kDa), H2A (~15 kDa), and H3 (~17 kDa). Western blotting using anti-Histone H3 and anti-Histone H4 antibodies detected single bands at approximately 17 kDa and 12 kDa, respectively. The probing with an anti-H2A antibody revealed a predominant band at ~30 kDa and a weaker band at ~15 kDa, corresponding to monomeric H2A. The higher molecular weight band at ~30 kDa likely represents SDS-resistant histone dimers (e.g., H2A–H2B) or oligomers, or histones with post-translational modifications. Together, these results confirm the presence of H3, H4, and H2A in the extracted fraction, supporting successful enrichment of core histones (**Figure 3B**).

### Planarian histone-enriched extracts possess antibacterial activity

The antibacterial activity of the histone preparation (1 µg) was tested against six bacterial species: *S. pneumoniae*, *S. aureus*, *E. faecalis*, *P. aeruginosa*, *K. pneumoniae*, and *E. coli*. Growth was monitored by OD_600_ measurements over time (**Figure 3C**). The degree of inhibition varied among species. *S. aureus* showed the most pronounced reduction in growth compared to untreated controls. In contrast, the Gram-negative species exhibited comparatively limited growth suppression under the same conditions. Based on this response, *S. aureus* was selected for further analysis (**Figure 3C**).

### Planarian histone-enriched extracts show dose-dependent inhibition of *S. aureus* and reduces bacterial viability

**Figure 4:**
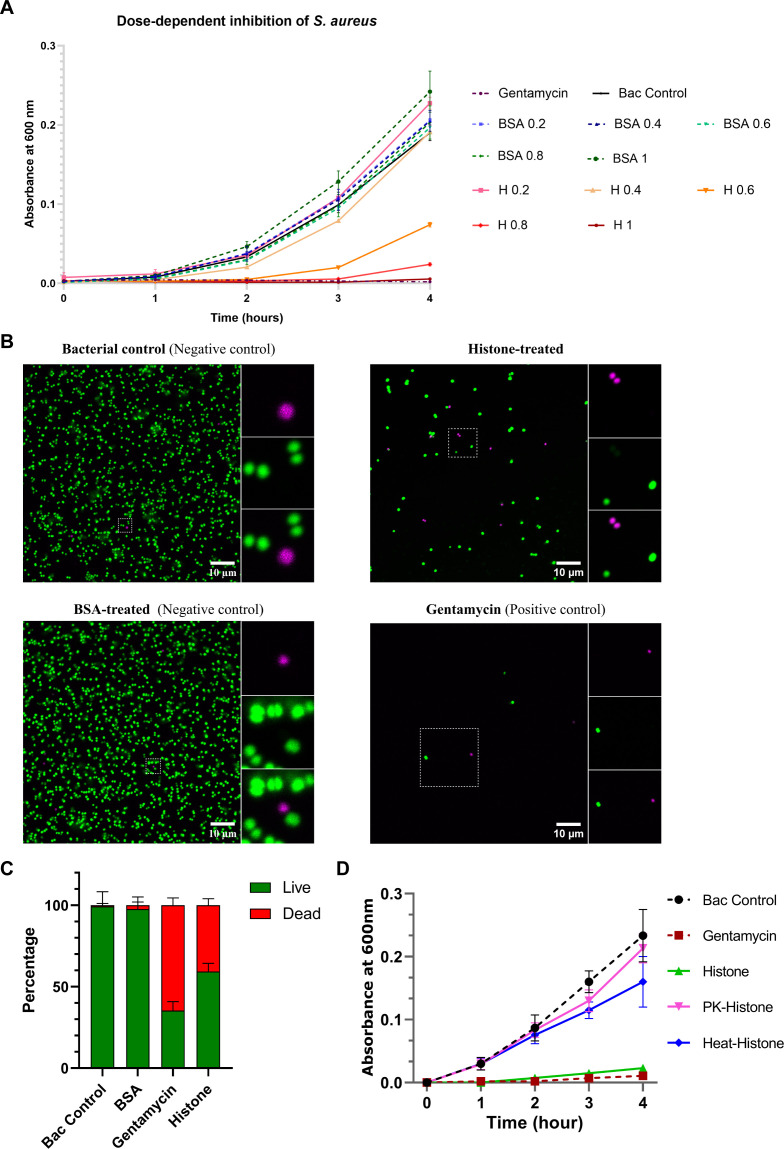
Antibacterial activity of planarian histone proteins against *S. aureus* and validation of protein dependency. **(A)** Growth kinetics of *S. aureus* following treatment with acid-extracted planarian histones (1 µg). Histone treatment showing a concentration-dependent reduction in bacterial growth monitored by measuring OD_600_ over time. Gentamycin served as positive control, BSA as protein control, and untreated bacteria as growth control. **(B)** Fluorescence microscopy of *S. aureus* after treatment with histones. Live/dead staining was performed using SYTO9 (green, live cells) and propidium iodide (magenta, dead cells). Untreated and BSA-treated bacteria showed predominantly green fluorescence, while histone-treated samples exhibited increased magenta signal, indicating loss of membrane integrity. Scale bar: 10 µm. Representative images are shown from three independent experiments (n = 3). **(C)** Quantification of live and dead bacterial populations based on fluorescence imaging. Data represent percentage of live (green) and dead (red) cells. Histone treatment significantly increased bacterial death compared to control and BSA-treated groups. Quantification was performed from three independent fields across three biological replicates. **(D)** Growth curves of *S. aureus* treated with native histones, Proteinase K–treated histones (PK-histone), and heat-denatured histones (heat-histone). Loss of antibacterial activity following Proteinase K digestion or heat denaturation indicates that growth inhibition depends on intact protein structure. Data represent mean ± SD from three independent experiments.

To assess whether inhibition was concentration-dependent, *S. aureus* was treated with increasing amounts of histone protein (0.2–1 µg), and growth was monitored by OD_600_ (**Figure 4A**). Gentamycin completely suppressed growth, whereas untreated and BSA-treated controls showed normal proliferation. BSA had no detectable inhibitory effect at any concentration tested. Histone treatment resulted in a concentration-dependent reduction in bacterial growth. Minimal inhibition was observed at 0.2 and 0.4 µg. Growth suppression became evident at 0.6 and 0.8 µg, and 1 µg produced marked inhibition, approaching the effect of gentamycin.

To determine whether growth inhibition was accompanied by reduced viability, bacteria were stained with SYTO9 and propidium iodide and analyzed by confocal microscopy (**Figure 4B**). Untreated and BSA treated samples consisted predominantly of SYTO9 positive cells, indicating high viability. In contrast, histone-treated samples showed increased PI staining, consistent with compromised membrane integrity and bacterial death. Gentamycin-treated bacteria showed extensive PI staining, confirming effective bactericidal activity. Quantitative analysis (**Figure 4C**) showed >98% viable cells in untreated and BSA-treated groups. In comparison, histone treatment increased the proportion of dead cells to approximately 33.5%, while gentamycin induced the highest level of cell death.

### Antibacterial activity of planarian histone-enriched extracts requires intact protein

To determine whether the antibacterial effect depended on protein integrity, histone-enriched preparations were treated with Proteinase K or subjected to heat denaturation prior to testing. Both protease digestion and heat treatment abolished antibacterial activity (**Figure 4D**). Treated samples displayed growth curves comparable to control groups, whereas untreated histones retained inhibitory activity. These findings indicate that the antibacterial effect depends on intact histone proteins.

## Discussion

In this study, we identified and characterized the core and linker histone repertoire of *S. mediterranea* and examined whether these proteins exhibit antibacterial activity. Sequence analysis confirmed that planarians possess a complete and structurally conserved set of histones, including canonical H2A, H2B, H3, H4, and H1, as well as a macroH2A variant. Phylogenetic analysis further showed that these proteins cluster by histone class rather than species, consistent with the strong evolutionary conservation of histones across metazoans.

While histones are primarily known for their role in chromatin organization, increasing evidence indicates that they can also function outside the nucleus. Histone-derived peptides and full-length histones have been reported to display antimicrobial activity in vertebrates and invertebrates, often acting through membrane disruption (Duong et al., 2025; Fu et al., 2020, 2020; Jodoin and Hincke, 2018; Marsman et al., 2024; Poirier et al., 2014; Sørensen and Borregaard, 2016; Wang et al., 2024; Zeng et al., 2024; Zhang et al., 2025). Mammalian histones can enhance AMP-mediated pore formation, hinder membrane recovery, depolarize the bacterial proton gradient, and subsequently enter the cytoplasm, where they restructure chromosomal DNA and suppress transcription (Doolin et al., 2020; Duong et al., 2020). Our findings extend this concept to planarians, an organism well known for its regenerative capacity but less explored in the context of innate immune effectors.

Acid-extracted histone preparations from *S. mediterranea* showed antibacterial activity in vitro, with the strongest effect observed against the Gram-positive bacterium *S. aureus*. In contrast, Gram-negative species were comparatively less affected under the same conditions. This pattern is consistent with the structural differences between Gram-positive and Gram-negative bacteria. The thick peptidoglycan layer and exposed teichoic acids of Gram-positive bacteria may facilitate electrostatic interactions with positively charged histone proteins, whereas the outer membrane of Gram-negative bacteria can act as a permeability barrier.

The inhibitory effect on *S. aureus* was concentration-dependent and accompanied by a measurable reduction in bacterial viability. Increased propidium iodide uptake in histone-treated samples suggests membrane compromise as a likely mechanism. Similar membrane-disruptive activity has been described for extracellular histones and histone-derived peptides in other systems (Hoeksema et al., 2016). Although we did not directly examine membrane ultrastructure or leakage of intracellular contents, the viability data are consistent with a bactericidal rather than purely bacteriostatic effect at higher concentrations.

Importantly, protease digestion and heat denaturation abolished antibacterial activity, indicating that the observed effect depends on intact protein structure. This argues against contamination by small non-proteinaceous molecules during extraction and supports a direct role for histone proteins themselves. At the same time, the preparation likely contains a mixture of histone subtypes. Whether the activity is driven by a specific histone, a particular domain, or a synergistic combination remains to be determined.

Planarians rely exclusively on innate immunity, lacking adaptive immune components. Their ability to maintain tissue integrity during regeneration despite constant environmental microbial exposure suggests the presence of efficient antimicrobial strategies. Extracellular or damage-released histones could represent one such mechanism. It is conceivable that histones released during tissue injury or cell turnover contribute to local antimicrobial defense, particularly in the early phases of wound response. Future work will be required to determine whether histones are actively secreted, passively released upon cell damage, or processed into smaller antimicrobial peptides in vivo.

Histones are highly cationic proteins and their antimicrobial activity is thought to arise primarily from electrostatic interactions with negatively charged bacterial membranes. Such interactions can destabilize membrane integrity and increase permeability, ultimately leading to bacterial death (Duong et al., 2025; Marsman et al., 2024). In some systems, histone-derived peptides have also been shown to penetrate bacterial cells and interfere with intracellular processes (Doolin et al., 2020; Duong et al., 2020). The increased propidium iodide staining observed in our viability assays is consistent with membrane disruption as a major mechanism underlying the antibacterial activity observed in this study.

Although our findings provide important insights into the antibacterial properties of planarian histone-enriched extracts, several limitations should be considered. The antibacterial assays were performed using histone-enriched nuclear extracts rather than purified individual histone proteins, and therefore the relative contribution of specific histone subtypes (e.g., H2A, H2B, H3, or H4) cannot be determined. It is also possible that multiple histones act synergistically to produce the observed antibacterial effects. Future studies using purified histones or recombinant proteins will be required to clarify the contributions of individual histone molecules.

In addition, the experiments reported here were performed under in vitro conditions. The concentrations required to inhibit bacterial growth in these assays may not directly reflect physiological levels in vivo. However, histones could reach locally elevated concentrations during processes that involve cellular disruption, such as tissue injury, infection, or regeneration. Planarians undergo continuous tissue turnover and extensive remodeling during regeneration, conditions that may transiently release nuclear proteins into the extracellular environment where they could contribute to antimicrobial defense. Future studies will be required to determine whether histone-mediated antibacterial activity contributes directly to host defense during infection or regeneration in vivo.

Beyond their direct antimicrobial effects, extracellular histones are increasingly recognized as damage-associated molecular patterns (DAMPs) released during cell death and inflammatory responses. In vertebrate systems, extracellular histones have been shown to mediate cytotoxicity, endothelial dysfunction, and organ injury in severe inflammatory conditions such as sepsis. For example, histones released during systemic inflammation can act as potent mediators of tissue damage and lethality, while their neutralization improves survival in experimental models (Xu et al., 2009). Similarly, cell death associated release of DAMPs, including nuclear components, can contribute to downstream organ dysfunction through effects on endothelial cells and inflammatory signaling pathways (Wu et al., 2024). These observations highlight the dual nature of histones as both antimicrobial effectors and inflammatory mediators. In this context, the antibacterial activity observed in planarian histone enriched extracts may reflect a conserved property of chromatin derived molecules that function at the interface of host defense and tissue damage responses.

In summary, our data show that *S. mediterranea* possesses a conserved histone repertoire and that histone-enriched preparations exhibit antibacterial activity, particularly against *S. aureus*. These findings broaden the functional perspective of histones in planarians and raise the possibility that chromatin proteins may contribute to innate immune defense beyond their canonical nuclear roles. Further studies aimed at dissecting the specific histone components and their mechanisms of action will help clarify the biological significance of this activity.

## Data Availability

The original contributions presented in the study are included in the article/supplementary material. Further inquiries can be directed to the corresponding author.
