# Correction: Chromatin-derived histone proteins from *Schmidtea mediterranea* function as innate antimicrobial effectors

**DOI:** 10.3389/fcimb.2026.1935991

**Published:** 2026-08-03

**Authors:** Devika Nampoothiri, Riddhi Bhardwaj, Nimish Deshpande, Prasad Abnave

**Affiliations:** 1 BRIC-National Centre for Cell Science, Savitribai Phule Pune University, Pune, Maharashtra, India; 2 Regional Centre for Biotechnology, NCR Biotech Science Cluster, Faridabad, India

**Keywords:** antibacterial, histone, infection, planarian, *Schmidtea mediterranea*

In the original article, there was a mistake in **Figure 1** as published. The domain of one of the proteins was wrongly labeled as “H2B” instead of “H2A” and one of the shorter “H2A” proteins was inadvertently omitted during figure preparation. The corrected **Figure 1** appears below.

Further more, in the article text, “The amino acid length ranges for “H2A” and “H2B” proteins were incorrectly reported as 120–135 and ~70–140 amino acids, respectively”. A correction has been made to **Results** Section, Subsection: *Identification and evolutionary conservation of planarian histones*. Paragraph Number: 2

The predicted H2A proteins were approximately 50–145 amino acids in length and contained the canonical H2A domain. One H2A variant possessed an extended C-terminal MACRO domain, consistent with a macroH2A-type protein. H2B proteins ranged from ~70–125 amino acids and carried the conserved H2B fold domain.

The authors apologize for this error and state that this does not change the scientific conclusions of the article in any way.

The original article has been updated.

**Figure 1:**
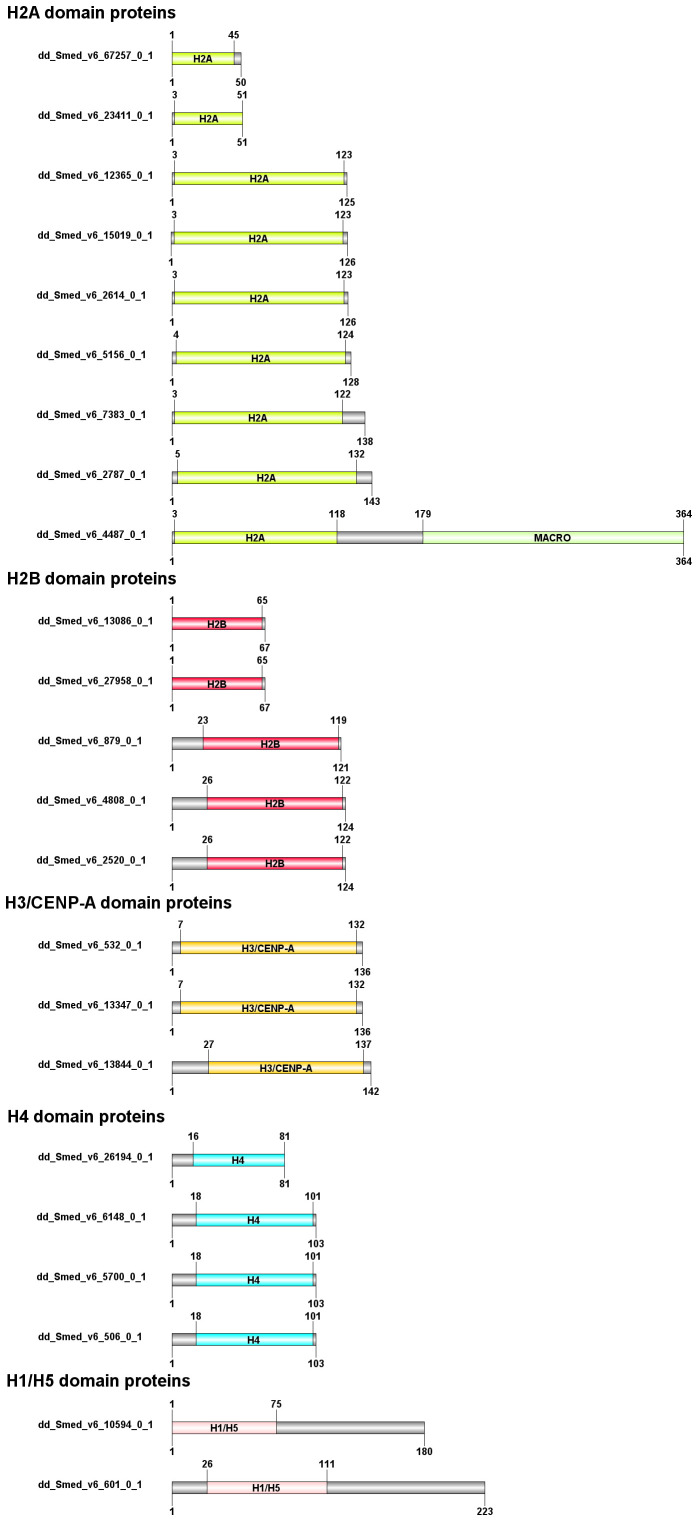
Identification of planarian histone proteins. Predicted histone proteins in *S. mediterranea* with H2A, H2B, H3/CENP-A, H4, and H1/H5 domains.

